# The utility of *Drosophila melanogaster* as a fungal infection model

**DOI:** 10.3389/fimmu.2024.1349027

**Published:** 2024-03-14

**Authors:** Chengetai D. Mpamhanga, Ilias Kounatidis

**Affiliations:** School of Life Health and Chemical Sciences, The Open University, Milton Keynes, United Kingdom

**Keywords:** *Drosophila*, model organisms, fungal diseases, WHO, FFPL, infection models

## Abstract

Invasive fungal diseases have profound effects upon human health and are on increase globally. The World Health Organization (WHO) in 2022 published the fungal priority list calling for improved public health interventions and advance research. *Drosophila melanogaster* presents an excellent model system to dissect host-pathogen interactions and has been proved valuable to study immunopathogenesis of fungal diseases. In this review we highlight the recent advances in fungal-*Drosophila* interplay with an emphasis on the recently published WHO’s fungal priority list and we focus on available tools and technologies.

## Introduction

### Fungal infections

The global impact of opportunistic fungal infections has gone underrecognized for a long time (1). However, with the increase in chronic and immunosuppressive health conditions including HIV/AIDS, cancer, cystic fibrosis and diabetes, antimicrobial therapies and invasive procedures that leave individuals vulnerable to opportunistic infections, the impact of these infections are becoming more apparent (1, 2). Fungi cause disease through direct infection of the host or through their secondary metabolites, mycotoxins, pigments that can contaminate the environment, food products and air (3). The disease burden ranges from superficial to invasive fungal infections and is estimated to be in the 100s of millions of patients per year, resulting in >1.5 million deaths/year (2, 4). These infections are caused by long recognised pathogens such as *Aspergillus fumigatus* and *Candida albicans* (5–7), neglected tropical diseases like eumycetoma (8, 9), and newly emerged pathogens, such as *Candida auris* (10, 11).

With the development of advanced molecular and cellular biology technologies, fungal pathogenicity and virulence factors are being studied in greater detail (12–14). However, the fungal threat continues to grow while the development of novel effective antifungal therapies remains inadequate (15, 16). As a result, in 2022 the WHO published the WHO fungal priority pathogens list, classifying 18 medically relevant fungal species as “Critical”, “High” or “Medium” priority, according to the perceived public health burden (2, 17).

### Model organisms

The use of model organisms is one of the technologies that has been developing over time and has become indispensable to investigating the nuances of host-pathogen interactions (18, 19). A cursory search of PubMed using the keywords “*Drosophila*” AND “fungi” yielded 8,617 results (1948 – 2023), with over a third (36.2%) of the publications having been released in the last decade alone. Seminal proof of concept studies in the 1990s and early 2000s, utilising wild-type and mutant *Drosophila* strains and fungi, provided a comprehensive framework for employing *Drosophila* in fungal research (20–25). Over the last decade, more extensive *Drosophila*-fungi work has taken place, leading to a better understanding of virulence, pathogenicity, and host immune responses (26–30).

### Purpose of review

This review sets out to provide a brief update on tools currently being applied to host-fungal interaction studies in *Drosophila* and highlight examples of research in the last 5 years with a focus on the WHO’s fungal priority pathogens list (17).

## *Drosophila* as the model organism

*Drosophila*, affectionately dubbed the biology “work horse”, has been used in fundamental biology, inbreeding and heredity studies since the early 1900s (27, 31) and has led to substantial contributions to our understanding of genetics, cellular biology, neurobiology and immunology (31, 32). Of note, the discovery of *Drosophila* Toll receptor nearly 3 decades ago elucidated the function of the analogous mammalian Toll-like receptor (TLR) pathway, which is indispensable to innate immunity (20; Lemaitre, 21, 26). *Drosophila* genome can be genetically manipulated, and genome-wide studies performed to determine genes crucial for survival and infection (27, 33). 75% of the genes responsible for human diseases have a homologue identified in Drosophila genome, an observation that highlights Drosophila’s suitability as a model for the study of mammalian disease conditions (34, 35).

**Figure 1:**
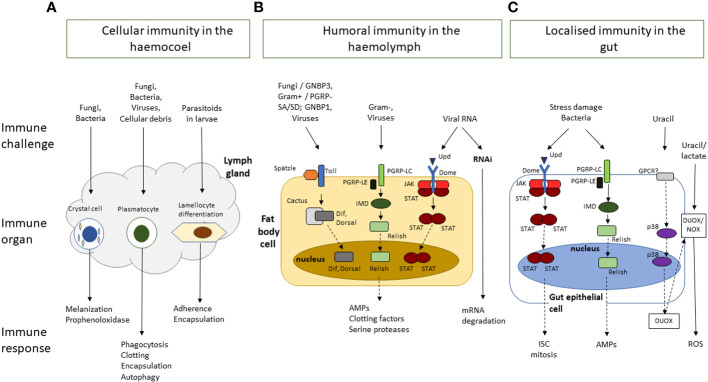
A simplified schematic overview of *Drosophila* melanogaster innate immune response to challenges by bacteria, viruses, fungi, or parasites, and to damage induced by stress or wounding. The immune responses are clustered by response type and location. **(A)** Cellular immunity in the hemocoel is mediated by crystal cells, plasmatocytes and lamellocytes, which are involved in, melanisation, phagocytosis, and encapsulation, respectively. **(B)** Humoral immunity in the haemolymph is mediated by the activation of signalling cascades in the Toll, Immune deficient (IMD) and JNK, JAK/STAT and mRNA degradation pathways following the recognition of pathogens and their virulence factors. It results in the production of a range of effector molecules including antimicrobial peptides (AMPs), clotting factors, and serine proteases. **(C)** The gut epithelium functions as an immune organ in response to pathogens and stress damage though the following responses: The JAK/STAT pathway responds to damage to increased proliferation of intestinal stem cells (ISC). The IMD pathway in response to bacteria presence in the gut leads to the production of AMPs. Finally bacterial-derived uracil induces the generation of reactive oxygen species (ROS) through the dual-oxidase (DUOX) and the NADPH oxidase (NOX). Dashed arrows represent additional steps involved in the signalling cascade, transcription, and translation, involved in the immune response.

*Drosophila* immunity relies on the innate immune system, made up of cellular and acellular components and regulatory pathways (**Figure 1**) (36, 37).These have been traditionally siloed into the humoral and cellular responses, though recent studies have shown that there is considerable crosstalk between the two branches (38, 39). *Drosophila* shares the following conserved innate immune pathways with vertebrates: the Toll and IMD NF-κB signalling pathways, the JNK pathway and the JAK/STAT pathway (40, 41). The Toll pathway responds to fungi and Gram-positive bacteria, while IMD responds to Gram-negative bacteria (40, 42). These pathways are activated by the recognition of pathogen antigens and host cell damage, and result in the production of effector molecules necessary for eliminating pathogens, autophagy and cellular repair, and immunomodulation as well as other *Drosophila*-induced Immune Molecules (DIMs) yet to be characterized fully (43, 44). These effectors have not yet been fully identified, but include antimicrobial peptides (AMPs), Boms (encoded by *Bomanins*), Daisho peptides (39, 45). AMPs are small, positively charged peptides that interact with hydrophobic regions of microbial cells walls and cause cell wall degradation and microbial death and are secreted into the haemolymph by the fat body (45, 46). In addition to AMPs, reactive oxygen species (ROS) are produced by Dual Oxidase (DUOX) and NADPH (Nox) at the epithelial cells (26, 32). The humoral response also provides protection against viral attack through RNAi and autophagy processes (36, 47).

*Drosophila* cellular immunity is mediated by the blood cell system which comprises of three differentiated populations. The major class of hemocytes are plasmatocytes which are considered equivalent to vertebrate macrophages. More than 90% of all hemocytes are plasmatocytes in every developmental stage of Drosophila (aside from the early-stage embryo) and they are responsible for the disposal of both microorganism and apoptotic cells. Another class are the crystal cells which are responsible for the melanisation in larvae. They contain the enzyme prophenoloxidase a key enzyme in melanin biosynthesis which is released upon rupture of the crystal cells. The third class refers to the lamellocytes, they are rare, but their number increases following oviposition by parasitoid wasps 48, 49). Haematopoiesis occurs at two different stages of ontogenesis: a first population derives from the head mesoderm during the stage of early embryogenesis, and a following second population that arises from the mesodermal lymph gland at a later stage of development (50 *).*

*Drosophila* antifungal immune responses rely heavily on the Toll pathway (37, 51). Toll signalling is activated by the binding of the surface antigen β-glucan to *Drosophila* recognition receptor Gram-negative binding protein 3 (GNBP3) and activates Toll through the activity of the Spätzle ligand and subsequent signalling cascade (51, 52). The Toll signalling cascade is also activated by the cleavage of the haemolymph serine protease Persephone by fungal enzymes contributing to the subsequent downstream activity of the Toll pathway (22, 53). The signalling cascade results in the production of specific AMPs, including Drosomycin, Daisho, Defensin and Metchnikowin, circulated in the hemolymph and the activation of the melanisation cascade to help resist the infection (42, 43, 45, 53).

## Application of *Drosophila* to human fungal pathogens

### Tools available for *Drosophila*-fungal studies

*Drosophila* is currently being utilised to investigate how medically relevant fungi interact with host immunity, and how they transition from colonization to infection (26–30). Wild-type and genetic mutant strains (e.g., Toll-deficient) are commercially available for distribution across the world from stock centres, such as the Bloomington Drosophila Stock Centre and Kyoto Stock Centre (54, 55). **Table 1** summarises *Drosophila* strains used in fungal research studies included in the current review. The Drosophila Genomics Resource Centre and ATCC are some of the suppliers who distribute Drosophila cell lines, like Schneider’s Drosophila Line 2 (S2) cell line, GFP-tagged cells, and cells from various organs for *ex vivo* studies (67, 68). These fly strains and cell lines are relatively inexpensive to purchase and maintain, increasing accessibility of the model (68). The model systems are infected or exposed to fungi, fungal secondary metabolites, and antifungal compounds to investigate these interactions (26, 55) via feeding, rolling over, or co-culture and in a standardised manner via needle pricking or microinjection, allowing for rapid inoculation of experimental groups (57, 58). Infection progression can be measured through survival, microbial load, mRNA quantification, melanisation and microscopy assays (55, 64). The efficacy and toxicity of antimicrobial compound screens can be measured in similar ways to determine their efficacy and toxicity (69, 70). Examples of microscopy techniques include confocal microscopy for visualising phagocytosis in fungi-stimulated plasmatocytes (71), electron microscopy for imaging effects of treatment on host cell morphology (35) and fungal burden (72). Immunofluorescence staining and bioluminescence allow visualisation of individual cell types in tissue, larvae or adult flies, and can be done through RNA *in situ* hybridization (30, 73, 74), intravital 2-d photon microscopy and reporter systems (GFP, lacZ) (62).

**Table 1:** List of *Drosophila* strains used in fungal infection studies.

Drosophila strain	Description	References
Wild type		
*w^A5001^*	White-eyed, wild-type immune system.	55
*w^1118^*	White-eyed, wild-type immune system.	43, 56;
Canton-S	Wild-type.	55
*y^1^w^1^*	Yellow body, white eyed.	55
*w^1,118^; y^1^*	Yellow body, white-eyed.	57
*Oregon^R^*	Red-eyed.	58, 59
Mutant		
*MyD88^c03881^*	Toll deficient.	55, 58
*MyD88-/-*	Toll deficient.	60, 61
*MyD88^kra1^*	Toll deficient.	51
*imd^shadok^*	Imd deficient.	51
*Bom^Δ55C^*	Bomanin deficient (elimination of 10 out of 12 Bom genes in the genome)	42, 43, 55, 62
*Tl^r632^/Tl^I-RXA^*	Toll-­deficient transheterozygote.	11, 63
*Tl[r3]/+)*	Heterozygous Toll deficiency.	64
*Rel^E20^*	White-eyed, Imd mutant.	56
*spz^6^*	Red-eyed, Toll mutant.	56
*w^1118^; np1-GAL4; DuoxRNAi*	GAL4 reporter system and dual oxidase (dDuox) knockout, wild type *w^1118^* background.	65
*FucTA^f03774^*	a piggyBac insertional mutant for the fucTA gene	66

The development of molecular techniques including DNA and RNA sequencing, RNAi gene silencing and CRISPR/Cas9 has advanced the field in leaps and bounds. Molecular techniques have made it possible to sequence the *Drosophila* genome (75); sequence coding and non-coding RNA and determine functionality through RNAi-based screening assays and gene silencing or overexpression (66, 76, 77). They facilitate quantification of messenger RNA (mRNA) or transfer RNA (tRNA) through quantitative PCR, reverse transcriptase PCR and modified-induced misincorporation tRNA sequencing (mim-tRNASeq) (78, 79). The gene editing tool CRISPR/Cas9 utilises guide RNA which matches with target gene (CRISPR) and CRISPR-associated protein 9 (Cas9), an endonuclease which helps to break the dsDNA and facilitate editing of the target gene (80) and it can be used for loss-of-function studies (12, 45, 81). Bioinformatics tools have been developed and adapted for genomic studies across microbial, *Drosophila* and human genomes and these allow for rapid screening of genomes and vast publicly available pathogen and fly data for potential targets for further study (79, 82). These software tools coupled with publicly accessible databases such as *Drosophila* Evolution over Space and Time (DEST), FlyRNAi (Drosophila RNAi Screening Center and Transgenic RNAi Project (DRSC/TRiP)) and FlyBase form a powerful computational component of the *Drosophila* tool kit (67, 82, 83).

With a focus on the WHO priority pathogens, we will highlight some examples of how *Drosophila* has been used to address key questions around host-fungal pathogen interactions, immunology and drug interactions with a focus on developments in the last five years.

### Critical priority group

The WHO classified *Cryptococcus neoformans*, *A. fumigatus*, *C. albicans*, and the recently emerged *C. auris* as “Critical” pathogens. *A. fumigatus* is a filamentous, airborne pathogen that causes invasive aspergillosis, in vulnerable populations, like cystic fibrosis patients (32, 84). *Drosophila* has been used to study *A. fumigatus* pathogenesis since as far back as 2005 (85, 86). In 2010, Chamilos and colleagues showed that pathogenicity of *A. fumigatus* strains in a Toll-deficient fly model was comparable to that in a mice model (25). Since then, the fly model has been used to study the virulence of *A. fumigatus* mating types, effects of fungal volatile organic compounds on larval development and comparative pathogenicity of *Aspergillus* strains collected from diverse sources (environmental, clinical, airborne) (29, 57, 87).

Fungi produce volatile organic compounds (VOCs) which are easily vapourised, carbon-based compounds made up of “alcohols, aldehydes, acids, ethers, esters, ketones, terpenes, thiols and their derivatives” (56, 57, 88). A study of the effects of *A. fumigatus* VOCs in *Drosophila* was carried out over a 15-day period, by co-culturing the fungi and fly model. Quantitative measurements of VOCs production showed that greater volumes of VOCs were secreted when *A. fumigatus* was cultivated at 37°C, than at the fly’s preferred incubation temperature of 25°C (57, 89). In addition, exposure to VOCs resulted in varying levels of toxicity, ranging from mild to severe, including reduced speed and success rate of metamorphosis or death of 3rd instar larvae (57, 90). Gas-chromatography mass spectrometry analysis of *A. fumigatus* VOCs detected isopentyl alcohol 1-octen-3-ol at the highest volume (57). A *Drosophila* infection model was subsequently used to show that 1-octen-3-ol caused greater sensitivity in male than female flies, resulting in reduced dehydrogenase activity and nitric oxide production, and increased ROS production (35). The connection to sex may explain the similar sensitivity distribution witnessed in humans postexposure to mould (35, 91). The *Drosophila* models used to investigate the immune response to mycotoxins and studies have shown that the Toll pathway and secreted Bomanins, specifically neuronal BomS6, mitigate the symptoms of *Aspergillus* mycotoxin exposure, namely restrictocin and verruculogen (55). This could contribute to our understanding of how mammalian immunity interacts with mycotoxins.

The study by Almaliki (57) investigated the effect of VOCs produced by a single *C. neoformans* strain and found that the VOCs of this severe pathogen caused more severe morphological effects and higher death rates than all the *A. fumigatus* strains that were tested (57). *C. neoformans* is a pathogenic yeast able to establish invasive infections in immunocompromised patients. It has been frequently associated with HIV/AIDS and accounts for as much as 15% of HIV-related deaths (17). A *Drosophila* S2 protein expression system has been used to produce and purify a recombinant cryptococcal protease, May1. This protease was used for further investigation as a target to identify compounds that could simultaneously inhibit the fungal protease and HIV-1 protease, which would provide dual protection and lower toxicity for HIV/AIDS patients (92). In the study by Almaliki and colleagues (57) regarding the toxicity of VOCs in *Drosophila, C. neoformans* VOCs cause significant delays in metamorphosis with eclosion rates of 44% compared to 80% for controls.

Contemporaneously with the growing number of studies in *Aspergillus*, *Drosophila* has been used to study *C. albicans*, one of the most common causes of candidiasis and blood stream infections (7, 93). In 2004, Alarco and colleagues published a Toll-deficient *Drosophila* model through which they demonstrated concordant *C. albicans* pathogenicity findings with mouse models, giving validity to the use of fly models (94). This was further corroborated by similar study in *Candida glabrata* mutant libraries (65, 95). Drosophila studies have been used to investigate host adaptation by *C. albicans*. Liu et al. (59) demonstrated the necessity of phosphate transporter, Pho48, in establishing candidiasis in the wild type *Oregon^R^* fly via infection with wild type *C. albicans* and *Pho48* null mutants (59). Null mutants were 3.5 times less likely to cause fly death than wild type strains 5 days post-infection (59). Glittenberg and colleagues (66) via a targeted genetic screening of 5698 RNAi lines described the protective impact of fucosylation in immune defence against *C. albicans*. A recent study in a *Bom* ^Δ55C^ fly model, (lacking the ability to produce the full range of Bomanin peptides) highlighted the ability of *Candida* sp. (including *C. albicans* and *C. auris*) to break down proline for energy, which may promote virulence. Moreover *C. albicans* mutants lacking the Proline UTilization genes *put1, put3* or *put1/put2* genes) showed reduced virulence compared to control fungal strain in the same fly infection model (62).

*Drosophila* infection models have been used to investigate the efficacy and toxicity of potential antifungal compounds. These include a Toll heterozygous Drosophila, *Tl[r3]/+*, used to test the naturally occurring compound, acid ellagic acid, against *C. albicans* where researchers showed statistically significant survival rates, and no toxicity at the proposed effective doses (64). Raj et al. (96) demonstrated a >70% survival rate of wild-type *Drosophila* infected with *C. albicans* when treated with *Syzygium samarangense* leaf extracted in methanol and dissolved in dimethyl sulfoxide. While the dosage applied to the *Drosophila* infection model was not specified, 50 mg of the *Syzygium samarangense* leaf extract was effective at clearing colonisation in an *ex vivo* porcine tongue and skin model, suggesting it could have utility as part of a topical treatment (96). *Drosophila* infection models can also be applied to antifungal studies for known compounds with the goal of reintroducing or repurposing old therapies. Clioquinol was administered orally to treat parasitic infections in the mid-1900s, however its use was discouraged due to perceived side effects (63, 97). Researchers investigated the antifungal efficacy and toxicity of Clioquinol in a Toll-deficient Drosophila model infected with *C. albicans* (63).

*Drosophila* has been utilised to investigate the novel pathogen *C. auris*. Wurster et al. (11) used a Toll-deficient mutant, *Tlr632/TlI-RXA* (which shows reduced AMP production and reduced phagocytic ability) to investigate the pathogenicity of *C. auris* clades identified at the time (Clade I-IV), and to determine the efficacy of azole to treatment (11). Their findings suggested that there was variability among the strains’ pathogenicity, though all strains were more pathogenic than *C. albicans* (11, 98).

### High priority group

Species of non-*C. albicans* (NCA) have been investigated using *Drosophila* models. While NCAs have typically accounted for a smaller fraction of candidiasis infections, their prevalence and resistance to azoles and echinocandins is on the rise (17, 53, 99). NCAs in the high priority group include *Nakaseomyces glabrata* (*C. glabrata*), *Candida tropicalis* and *Candida parapsilosis*.

In 2018, researchers harnessed CRISPR/Cas9 for the targeted deletion of individual *Drosophila Bomanin* genes to determine their immunoprotective role against *C. glabrata* (43). Using *in vivo* and *ex vivo* infection models, they demonstrated that *Bomanin* genes do not act in tandem and the short-form Bom peptide was immunoprotective against *C. glabrata* on its own (43). They showed that flies lacking 10 out of the 12 *Bomanin* genes (42) were as susceptible to infection as Toll-deficient flies, highlighting the importance of Boms in host immunity (43). Studies in *Drosophila* cell lines have been used to identify mechanisms by which *C. glabrata* evades innate immunity strategies, like AMPs and ROS, and potential drug targets. A study by Kounatidis and colleagues showed that *C. glabrata ADA2* gene is essential for the pathogen to resist oxidative stress as the *ADA2* knockout yeast could only grow in flies with suppressed ROS, while overexpressing *ADA2* promoted *C. glabrata* growth and resulted in lower host survival rates (65). The role of the potassium transporter *C. glabrata* TRK1 was elucidated through infection of *MyD88* and *Bom* ^Δ55C^ *Drosophila* strains with wild type and *C. glabrata trk1* knockout (60). Loss of *TRK1* gene resulted in cell wall modifications and reduced virulence within the host environment, in a potassium concentration dependent manner (60).

*C. parapsilosis* is associated with neonatal infections in addition to candidemia and candidiasis in immunosuppressed patients (17, 53). The Toll pathway has been shown to be crucial for *Drosophila* survival when infected by *C. parapsilosis* (which was not the case for *Persephone* protease), by comparing the susceptibility of wild type and mutant *MyD88−/−* flies to *C. parapsilosis* (53).

In addition, the high group includes Mucorales, *Fusarium* sp. *Histoplasma* sp. and eumycetoma causative agents (17). Mucorales are a large group of ubiquitous, filamentous fungi, frequently found in soil, which can cause infections ranging from mild to invasive (100, 101). The Order includes genera like *Rhizopus*, *Mucor* and *Lichthiemia* (101). Building on previous preexposure studies that showed the utility of *Drosophila* in Mucorales studies, Wurster and colleagues showed that exposing three Mucorales, *Rhizopus arrhizus, R, pusillus*, and *Mucor circinelloides*, to the triazoles isavuconazole and voriconazole, triggered hypervirulence in the fungi, resulting in lower survival rates in a Toll-deficient model (*Tlr* ^632^ */TlI* ^-RXA^). This was a significant finding as it could explain infections arising in patients undergoing prophylaxis or treatment with isavuconazole (102). While the number of Mucorales tested was small, this gives some insight into this treatment challenge. This is in contrast with *A. fumigatus*, which often occupies the same niche and is managed in a similar way, but does not develop isavuconazole-induced hypervirulence (102).

The *Fusarium solani* species complex, includes *F. solani sensu stricto*, *F. falciforme* and *F. keratoplasticum*, and they are major opportunistic fungal pathogen, capable of causing keratitis (58). A screen of 42 environmental and clinical isolates from South India revealed that all isolates were intrinsically resistant to first-generation azoles and susceptible to imidazole, which contributes to treatment challenges (58). Survival assays comparing Oregon-R wild type and *Myd88* mutant flies, infected with 6 Fusarium sp. found that MyD88 is required to mount an effective Toll defence against all *Fusarium* strains (58). Homa et al. (58) also showed that *Fusarium* virulence was distinct at strain level (58). Subsequently, Cohen et al. found that survival rates following Daisho peptide knockout also varied among *Fusarium* species (51).

### Medium priority group

The lower priority category has the highest number of pathogens including *Scedosporium* sp, *Lomentospora prolificans*, *Coccidioides* sp, *Pichia kudriavzeveii* (*Candida krusei*), *Cryptococcus gattii*, *Talaromyces marneffei*, *Pneumocystis jirovecii* and *Paracoccidioides* sp (17). These pathogens have the lowest relative global incidence and mortality rates, but still have substantial impacts (2). One of these pathogens, *T. marneffei* is a thermally dimorphic fungal pathogen localised to South and Southeast Asia (61, 103). It is found in the environment and Bamboo rats and can be inhaled and establish severe invasive infections in humans and animals (61). Its prevalence is not fully known due to limited surveillance and diagnostics and though mortality rates can be as high as 30%, few host-pathogen interaction studies have been performed (103, 104). Qu et al. (61) used the *MyD88 −/−* fly model to investigate the significance of the *T. marneffei* mating type on virulence in 107 clinical, Bamboo rat and environmental samples. They demonstrated that the mating type (MAT1-1 or MAT1-2) did not have an impact on flies survival upon infection, despite the fact that MAT1-2 isolates were overabundant across the entire sample population (61).

## Perspective and future opportunities

The utility of *Drosophila* infection models in fungal research has been substantiated through the development of a good range of infection models and relevant findings. In spite of challenges and limitations around selecting the most suitable animal model, the extensive research work carried out in *Drosophila* over the last decade shows that this model is suitable. Fitting this extensive work within the boundaries of a Mini review article was a key challenge in setting up this review, therefore the WHO fungi prioritisation proved valuable into narrowing down the relevant content. While *Drosophila* presents a useful and relatively simple tool, subsequent investigations in other animal models are often required and should be considered to further corroborate findings, prior to reaching any general conclusions. Future work could focus on further characterisation of effector molecules (many of them have yet unknown function), on the role of innate immune mechanisms on immune memory adaptions, and on the use of *Drosophila* as a preclinical model on screening for antimicrobial efficacy against the fungal pathogens highlighted by WHO (17).

## Author contributions

CM: Writing – review & editing, Investigation, Writing – original draft. IK: Writing – review & editing, Conceptualization, Supervision, Validation.
